# On-Site Fluorescent Detection of Sepsis-Inducing Bacteria using a Graphene-Oxide CRISPR-Cas12a (GO-CRISPR) System

**DOI:** 10.1021/acs.analchem.3c05459

**Published:** 2024-01-30

**Authors:** Tom Kasputis, Yawen He, Qiaoqiao Ci, Juhong Chen

**Affiliations:** † Department of Biological Systems Engineering, Virginia Tech, Blacksburg, Virginia 24061, United States; ‡ Department of Bioengineering, University of California, Riverside, California 92521, United States

## Abstract

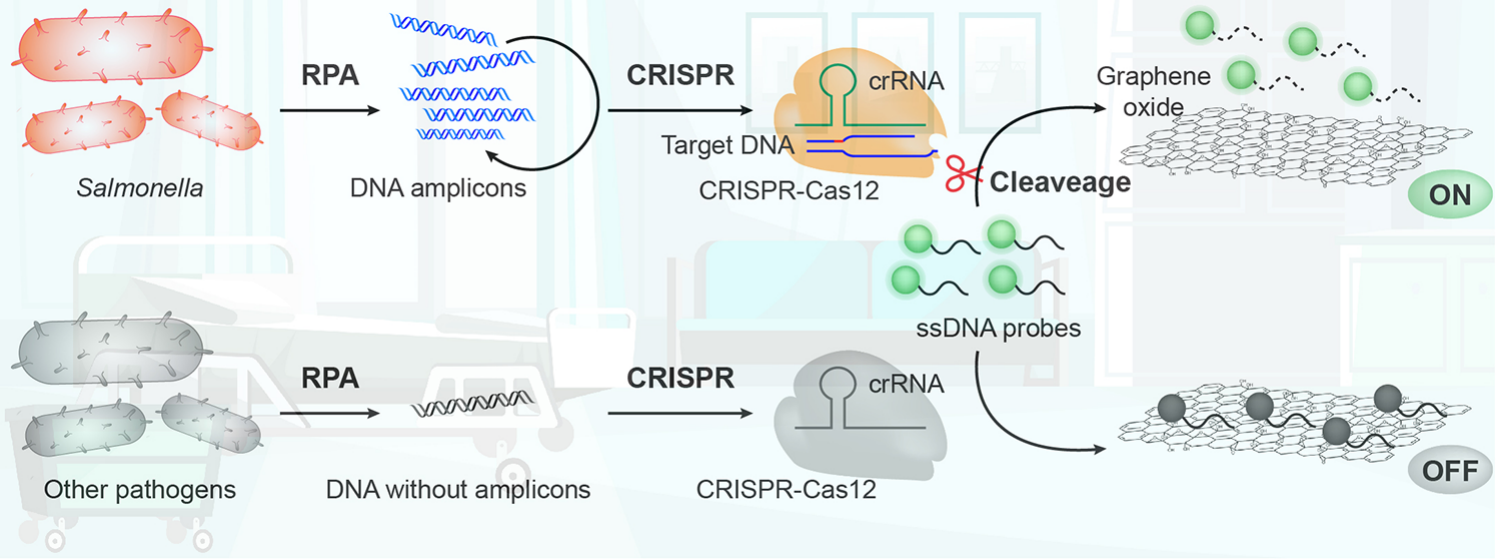

Sepsis is an extremely dangerous medical condition that emanates from the body’s response to a pre-existing infection. Early detection of sepsis-inducing bacterial infections can greatly enhance the treatment process and potentially prevent the onset of sepsis. However, current point-of-care (POC) sensors are often complex and costly or lack the ideal sensitivity for effective bacterial detection. Therefore, it is crucial to develop rapid and sensitive biosensors for the on-site detection of sepsis-inducing bacteria. Herein, we developed a graphene oxide CRISPR-Cas12a (GO-CRISPR) biosensor for the detection of sepsis-inducing bacteria in human serum. In this strategy, single-stranded (ssDNA) FAM probes were quenched with single-layer graphene oxide (GO). Target-activated Cas12a *trans*-cleavage was utilized for the degradation of the ssDNA probes, detaching the short ssDNA probes from GO and recovering the fluorescent signals. Under optimal conditions, we employed our GO-CRISPR system for the detection of *Salmonella* Typhimurium (*S*. Typhimurium) with a detection sensitivity of as low as 3 × 10^3^ CFU/mL in human serum, as well as a good detection specificity toward other competing bacteria. In addition, the GO-CRISPR biosensor exhibited excellent sensitivity to the detection of *S*. Typhimurium in spiked human serum. The GO-CRISPR system offers superior rapidity for the detection of sepsis-inducing bacteria and has the potential to enhance the early detection of bacterial infections in resource-limited settings, expediting the response for patients at risk of sepsis.

## Introduction

Sepsis poses a significant and concerning threat to healthcare, particularly among newborns and infants. Neonatal sepsis is often characterized by nonspecific signs and symptoms, requiring early and accurate diagnosis of infection to improve clinical outcomes. This condition can lead to life-threatening complications and death if not promptly treated and diagnosed.^1^ In fact, nearly four million deaths are attributed to neonatal sepsis infections annually, most commonly in resource-poor areas.^2^ Bacterial agents are the most common potential causes of neonatal sepsis. This occurs by bacterial infections triggering a cascade of inflammatory responses upon entering the bloodstream, leading to sepsis-vulnerable persons.^3^ Quick intervention upon the determination of bacterial infection is critical in sepsis prevention. Therefore, rapid diagnostics are necessary to overcome the limitations of the traditional culture methods.

Polymerase chain reaction (PCR) provides much quicker results compared with the traditional culture-based plate counting methods. However, PCR-based methods typically require specialized equipment and trained personnel, hence they are typically only carried out in a laboratory.^4^ Additionally, these may be limited for expansive diagnostics in resource-poor areas where neonatal sepsis is the most prevalent. As a result, there is growing urgency for rapid on-site bacterial detection methods that can detect bacterial infections earlier and prevent the dangers that occur with sepsis. On-site detection methods should provide quick and accurate detection of sepsis-related bacteria, enabling timely interventions for the treatment of bacterial infections and sepsis prevention. Many point-of-care (POC) biosensors have been developed to address the urgency of on-site bacteria detection. These biosensors often utilize surface-enhanced Raman spectroscopy (SERS),^5^ surface plasmon resonance (SPR),^6^ or electrochemistry.^7^ While these may provide more direct screening of bacterial infections in hospitals, these sensors are often complex and costly to manufacture or require extensive preprocessing steps. Simpler options such as optical biosensors^8^ and lateral-flow assays have been developed, however, often lack the necessary sensitivity and specificity for reliable detection.^9^ Hence, the ideal POC biosensor to detect sepsis-inducing bacteria should be affordable, reproducible, sensitive, and reliable.^10^

Clustered-regularly interspaced short palindromic repeats (CRISPR)-Cas systems, originally discovered as a gene-editing tool, provide unique and promising tools for POC biosensors due to their highly specific target recognition properties.^11^ In recent years, CRISPR-Cas systems have been employed for numerous innovative biosensors.^12−16^ Specifically, CRISPR-Cas12a systems offer particular advantages in DNA detection compared with other POC biosensors due to their simplicity of use and high target specificity.^17−19^ In Cas12a systems, a specifically designed CRISPR guide RNA (crRNA) binds to the Cas12a enzyme to form a Cas12a/crRNA complex. Upon hybridization of the protospacer adjacent motif (PAM) site from the target DNA to the crRNA, the complex is activated by target recognition. This results in cleavage of the double-stranded DNA (dsDNA) targets. Exceptionally, the activated Cas12a/crRNA complex possesses nonspecific *trans*-cleavage activity as well. Upon target initiation, the CRISPR complex degrades any surrounding single-stranded DNA (ssDNA) into short fragments. This *trans*-cleavage mechanism has been leveraged for the creation of many relevant CRISPR-Cas12a-based biosensors,^20,21^ including for the detection of parasitic infections,^22^ bacterial infections,^23^ drug-resistant bacteria,^24^ and more. These systems often utilize fluorescence as a reliable signal transduction method.^25,26^^27^ While CRISPR-based fluorescent biosensors have successfully been developed for the detection of bacteria, complicated probe designs and expensive fluorescent quenchers have hindered the true portability and affordability for POC detection.^28−30^ Additionally, standard fluorescent quencher (FQ) probes can cause steric hindrance, which can lower the *trans*-cleavage activity thus lowering sensitivity.^31^ Recently, nanomaterials have demonstrated the ability to offer simpler and more accessible options for fluorescent quenching.^32^

Single-layer graphene oxide (GO) has been shown to exhibit extremely specific distance-dependent fluorescent quenching.^33^ In the close proximity of graphene oxide, an excited fluorophore can transfer its energy to the graphene oxide sheet, suppressing the fluorescence signals.^34^ Additionally, ssDNA can attach to the GO surface through π–π stacking interactions between nucleic acids and carbon atoms in the graphene lattice, resulting in a noncovalent binding force.^35^ While effective for longer ssDNA, the binding force is extremely weak for short DNA strands.^36^ In recent years, several fluorescent biosensors have been designed relying on precise control of ssDNA fluorescent probes and single-layer GO.^37^ Additionally, GO-induced quenching has been shown to exhibit superior efficiency in fluorescence quenching when compared to similar nanomaterials, such as gold nanoparticles.^32^ Therefore, the simplicity and availability of GO offer improved affordability and effectiveness in comparison to standard fluorescence-based biosensors.^38^

Herein, we present a graphene-oxide CRISPR-Cas12a (GO-CRISPR) system for the rapid and sensitive detection of sepsis-inducing bacteria on-site. We validate our sensor with the detection of *Salmonella*. Nontyphoidal *Salmonella* represents one of a few select pathogens that cause the majority of sepsis infections.^39,40^ Recombinase polymerase amplification (RPA) is applied for the isothermal amplification of bacterial DNA, circumventing the reliance on complicated machinery for gene amplification. The CRISPR-Cas12a system then provides superior accuracy and sensitivity for the recognition of *Salmonella* DNA for target-specific *trans*-cleavage of the fluorescent probes.^41^ The GO then quenches only undegraded probes. Our system presented a detection limit of *S*. Typhimurium as low as 3 × 10^3^ CFU/mL in human serum within an hour. The developed sensing mechanism offers new advantages for the on-site detection of bacteria. We envision that this detection assay will be expanded to other sepsis-inducing pathogens to further combat the fight against sepsis.

## Materials and Methods

### Materials and Reagents

Bacteria strains of *Salmonella* Typhimurium *(ATCC 10428)*, *Salmonella* Newport, *Salmonella* Tennessee, *Salmonella* Seftenberg, and *Staphylococcus aureus* (ATCC 13565), and *Listeria monocytogenes* (ATCC 19115) were kindly provided by Kim Waterman from the Department of Food Science and Technology at Virginia Tech. Other bacterial strains were purchased from the American Type Culture Collection (Manassas, VA), including *Escherichia coli* K12 (ATCC 25404) and *Bacillus subtilis* (ATCC 23857). Graphene oxide (GO) was purchased from Cheap Tubes Inc. (Grafton, VT). PCR amplification was performed using a Q5 High-Fidelity PCR kit (New England Biolabs, Ipswich, MA). All nucleic acids including CRISPR-RNA (crRNA) were purchased from Integrated DNA Technologies (Coralville, IA). The recombinase polymerase amplification (RPA) was performed using the TwistAmp Basic Kit purchased from TwistDX (Maidenhead, United Kingdom). The fluorescent analysis was carried out using an Agilent BioTek Synergy H4 Hybrid Microplate Reader from Fisher Scientific (Waltham, MA). AsCas12a nucleases were expressed and purified using custom pET-based expression vectors following previously reported methods.^12^

### PCR Amplification of *Salmonella*

*Salmonella* DNA (274 bp) from the *invA* gene was amplified from *Salmonella* using a Q5 High-Fidelity PCR reaction system The total reaction volume (50 μL) contained PCR Master Mix (25 μL), the forward and reverse primers (100 μM, 2.5 μL each) and *Salmonella* culture (1 μL), and RNase-free water. The PCR reaction was performed for 35 cycles in a Bio-Rad T100 Thermal Cycler. The PCR products were confirmed using agarose electrophoresis analysis and cleaned with the Monarch DNA Cleanup Kit.

### Characterization of Fluorescent Quenching

Single-layer GO at various concentrations (0–2000 μM) was prepared in 1 × NEB Buffer 2.1. The ssDNA-FAM probes (100 nM, 10 μL) were quenched by the GO solutions (10 μL) at final concentrations ranging from 0 to 200 μM. The solutions were mixed in the BioTek Synergy H4Microplate Reader at room temperature for 10 min to allow for binding between the ssDNA-FAM probes and the GO. The fluorescence intensities were measured with an excitation of 485 nm and emission wavelengths ranging from 510 to 600 nm.

### GO-CRISPR Detection of *Salmonella* DNA

The GO-CRISPR detection of *Salmonella* DNA consisted of the CRISPR-Cas12a *trans*-cleavage reaction and subsequent fluorescent quenching using GO. The CRISPR-Cas12a reaction (90 μL) contained the *Salmonella* DNA (10 μL), ssDNA-FAM probes (100 nM, 10 μL), Cas12a (1.2 μM, 10 μL), crRNA (1.4 μM, 10 μL), and 1 × NEB Buffer 2.1. The reaction mixture was incubated at 37 °C for 30 min to allow for sufficient *trans*-cleavage of the probe. Directly following the CRISPR reaction, GO (1400 μM, 10 μL) was added to the solution. The solution was then mixed at room temperature for 10 min, and the fluorescent intensity was measured with the same excitation and emission as previously reported.

### Preparation of Bacterial Cultures

The stock cultures of Salmonella Typhimurium, Salmonella Newport, Salmonella Tennessee, Salmonella Seftenberg, *Staphylococcus aureus*, *Escherichia coli*, *Bacillus subtilis*, *Listeria monocytogenes*, and *Vibrio cholerae* were grown at 37 °C for 18 h in LB broth. To enumerate bacterial concentrations, the bacteria were plated on LB agar plates at 37 °C for 20 h. The bacterial cultures were then diluted 10-fold into various concentrations (10^1^ through 10^7^ colony-forming units (CFU)/mL) for further applications.

### RPA Amplification of Bacteria

PA reactions were performed with the TwistAmp Basic Kit following the standard manufacturer protocol. The total RPA reaction volume (50 μL) contained Primer Free Rehydration Buffer (29.5 μL), the forward and reverse primers (10 μM, 2.4 μL each), MgOAc (280 mM, 2.5 μL), a lysed bacterial culture (1 μL), and RNase-free water. The reaction mixture was incubated at 39 °C for 25 min. The amplified product (2.5 μL) was then diluted into 1 × NEB Buffer 2.1 (7.5 μL). This 10 μL solution was then reacted with the GO-CRISPR system following the previously described methods.

### Preparation of Spiked Human Serum Samples

To evaluate the GO-CRISPR system to detect sepsis-inducing bacteria in human serum (10 μL), *S*. Typhimurium at various concentrations of 10^2^ to 10^7^ CFU/mL were spiked in normal human serum (990 μL) obtained from Thermo Fisher Scientific. The human serum was then diluted 1:10 in phosphate-buffered saline (PBS). The samples were then lysed for 15 min at 95 °C. Finally, these solutions were amplified using RPA and evaluated with the GO-CRISPR system for the detection of *Salmonella* in human serum. A sample without any spiked bacteria was used as a negative control.

## Results and Discussion

**Figure 1:**
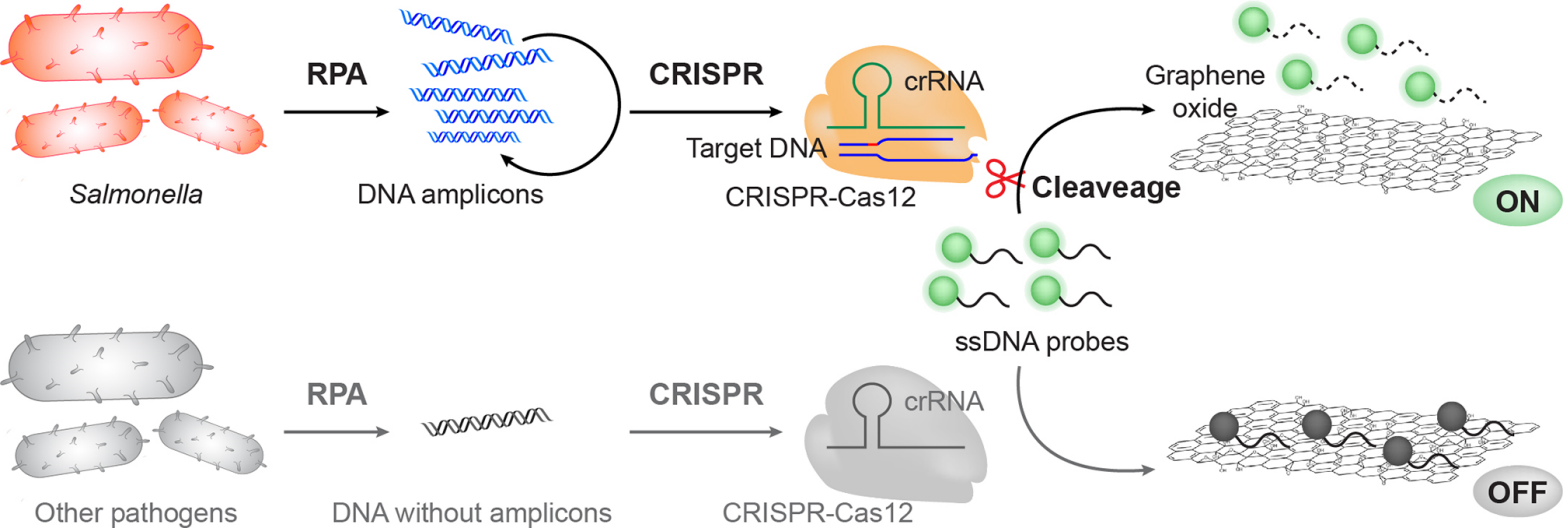
Schematic illustration of *Salmonella* detection using the GO-CRISPR system. The *invA* gene from *Salmonella* is amplified using isothermal recombinase polymerase amplification (RPA). The amplified *Salmonella* target DNA is then reacted with specifically designed CRISPR systems and ssDNA-FAM probes. In the presence of the target DNA, the CRISPR system is activated, initiating robust degradation of the probes. The degraded probes cannot bind to the surface of the GO, resulting in a fluorescent signal for visual detection of *Salmonella*.

The detection principle of the GO-CRISPR system is illustrated in Figure 1. First, *Salmonella* DNA undergoes isothermal amplification using RPA. We specifically target a short sequence of the *invA* gene, chosen for its broad conservation across *Salmonella* serovars and ideal for the detection of *Salmonella* using genetic methods.^42^ The amplified *Salmonella* target DNA is then reacted with specifically designed crRNAs, Cas12a proteins, and ssDNA-FAM probes.^32^ In the presence of the target DNA, the CRISPR system is activated, initiating a robust degradation of the probes through the nonspecific *trans*-cleavage mechanism and dissecting the 30-nucleotide (nt) probes into numerous shorter ssDNA strands. Following the CRISPR reaction, the solution is mixed with GO, enabling quenching of undegraded probes via π–π stacking of the longer ssDNA. However, due to the weak interactions between GO and short ssDNA, the degraded probes are unable to bind to the graphene oxide, resulting in a fluorescent signal for visual detection. Remarkably, this method facilitates a visual readout within one h, achieving both sensitive and specific detection of *Salmonella*.

### Characterization of the GO-CRISPR System

**Figure 2:**
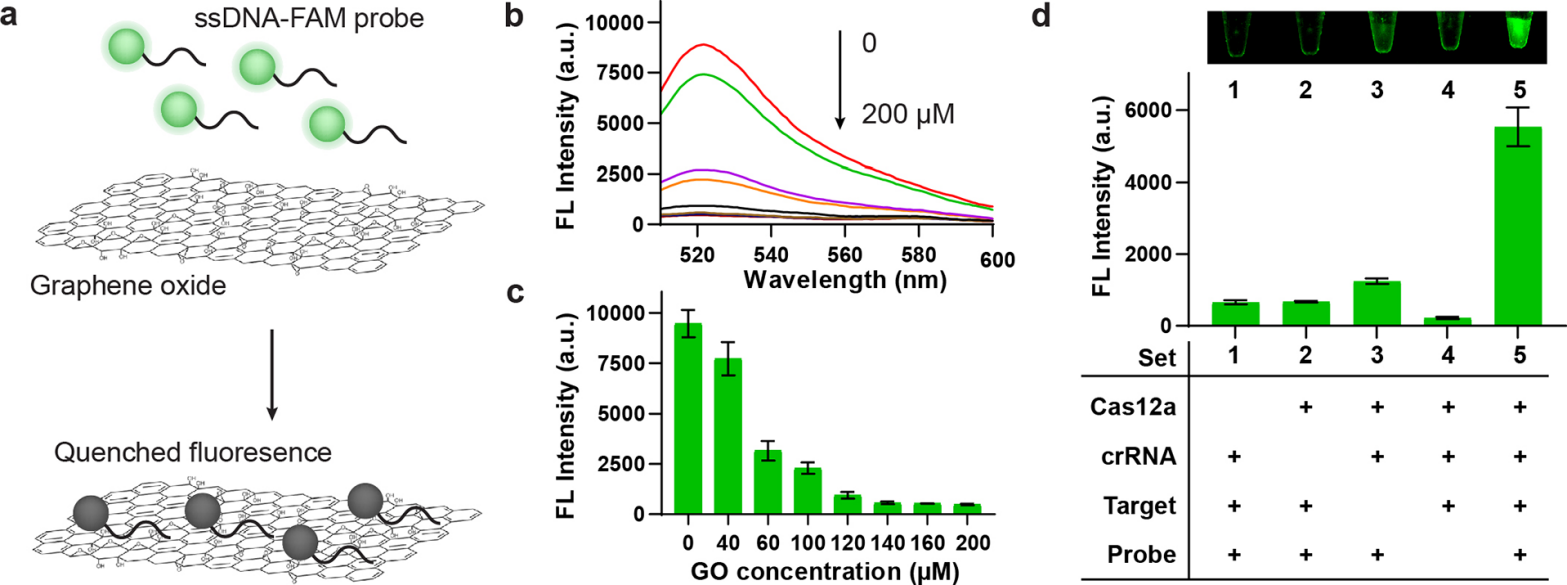
Characterization of the GO-CRISPR system. (a) Schematic illustration of distance-dependent GO fluorescent quenching. The ssDNA on the fluorescent probes attaches to the GO surface through π–π stacking interactions. The fluorescence is quenched in close proximity to the GO. (b) Fluorescent spectra of varying concentrations of GO from 510 to 600 nm. (c) Fluorescent intensity at 520 nm of varying concentrations of GO. (d) Fluorescent image and intensity for the GO-CRISPR system feasibility analysis.

To guide our GO-CRISPR system toward optimal sensitivity and robustness, the parameters governing the GO quenching interaction must be meticulously optimized. We designed a 30-nt ssDNA probe consisting of a FAM molecule and a poly(A20) tail (Table S1). The ssDNA tail was the optimal length for previously characterized noncovalent interactions between nucleotide bases and GO for π–π stacking, therefore resulting in sufficient fluorescent quenching (Figure 2a).^43^ Henceforth, our first aim was to determine the concentration of GO necessary for achieving complete fluorescent quenching. This is an important precursor to the GO-CRISPR assay as an insufficient GO concentration may produce high background noise, while excessive oversaturation of GO could compromise the fluorescence output and devalue the results. Therefore, we held the concentration of the ssDNA-FAM probe constant (10 nM) to ascertain the optimal GO/probe ratio. We analyzed a range of GO (0–200 μM) mixed with probes (Figure 2b). A large fluorescent signal at 520 nm, approximately 9000 au, was observed for the fully unquenched probes. As the concentration of GO increased, the fluorescent signal steeply decreased to approximately 1000 au at 120 μM before leveling off around 600 a.u at 140 μM (Figure 2c). Therefore, we determined that GO at 140 μM was the optimal concentration to achieve complete fluorescent quenching.

Next, we then evaluated the GO-CRISPR assay within a 100 μL system (140 μM GO, 10 nM probe, and a saturated amount of Cas12a, crRNA, and *Salmonella* target DNA). The fluorescent signal was generated exclusively in the presence of all CRISPR reagents with the target gene. Therefore, we determined that the Cas enzyme, crRNA, probe, and target are all necessary to elicit a discernible fluorescent readout (Figure 2d). Notably, there was an increase in the background signal when the crRNA and Cas12a were present with the probe. This could be due to interference with the graphene oxide caused by the protein complex.^44^ However, only the full GO-CRISPR system displayed a visual fluorescent signal, affirming that the presence of *Salmonella* DNA can be reliably detected by using our fluorescent assay.

### Optimization of Experimental Parameters

**Figure 3:**
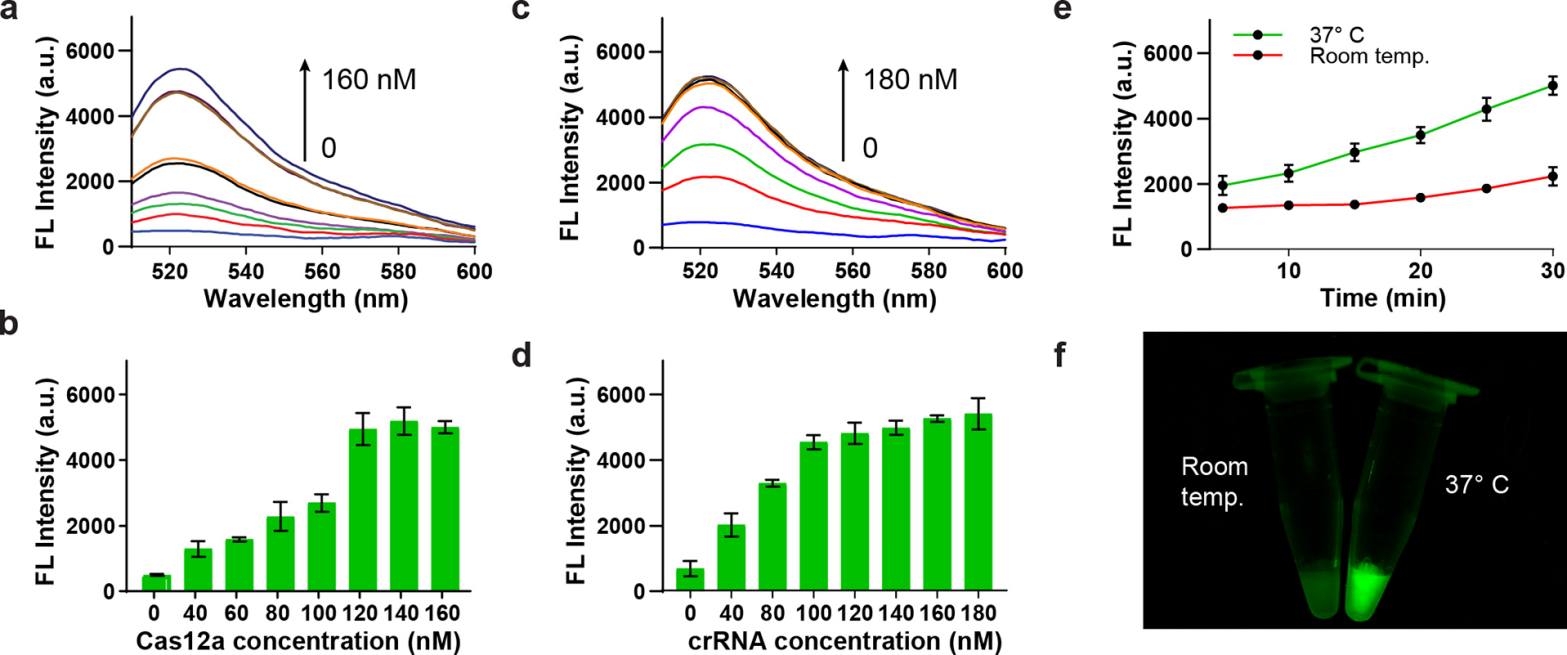
Optimization of the GO-CRISPR system. (a,b) Fluorescent spectra and intensity at 520 nm of varying concentrations of Cas12a. (c,d) Fluorescent spectra and intensity at 520 nm of varying concentrations of crRNA. (e,f) Fluorescent intensities at 520 nm and image at 30 min of the GO-CRISPR system at 37 °C and room temperature.

After establishing the feasibility of the GO-CRISPR system, we carefully optimized several crucial parameters for the *trans*-cleavage reaction. Our initial focus of this study was to determine the necessary concentration of the Cas12a enzyme for maximum *trans*-cleavage efficiency. To this end, *Salmonella* DNA (30 nM), FAM probes (10 nM), excess crRNA, and varying concentrations of Cas12a (0–160 nM) were reacted for 30 min. As shown in Figure 3a,b, the fluorescent intensity exhibited a gradual increase as the Cas12a concentration increased from 0 to 100 nM, reaching a fluorescence level of approximately 3000 au. The fluorescent intensity then leveled off around 5000 au from 120 to 160 nM. Beyond 120 nM, no discernible fluorescent difference was observed. Therefore, the reaction reached saturation at this concentration, and we identified Cas12a at 120 nM as the optimal concentration for the GO-CRISPR system.

Second, we optimized the concentration of crRNA for the GO-CRISPR system. Prior investigations into CRISPR-Cas12a systems have revealed that the optimal ratio of crRNA to Cas12a fell within the ranges 1:1 and 2:1. Additionally, an excess of crRNA has been shown to marginally inhibit CRISPR reactions.^45^ Hence, the determination of the ideal crRNA is pivotal for achieving the optimal sensitivity of our GO-CRISPR system. We systematically tested various concentrations of crRNA (0–180 nM, with crRNA to Cas12a ratios spanning from 0:1 to 2:1). These were reacted with Cas12a (120 nM), *Salmonella* DNA (30 nM), and FAM probes (10 nM). The fluorescent intensity sharply increased from 0 to 100 nM, followed by a subtle increase above 100 nM (Figures 3c,d). At the concentration of 140 nM, the fluorescence signal was seven times stronger than the background, and further increments in concentration did not yield significant enhancements in fluorescence. Consequently, we identified the optimal concentration of crRNA as 140 nM, where the fluorescent intensity plateaued around 5000 au

Lastly, we aimed to validate the optimal temperature for the reaction. It is well understood that the temperature is very important for enzymatic reactions. Previous CRISPR-Cas12a assays have demonstrated peak efficiency at 37 °C.^46^ Our investigation sought to determine the difference in fluorescence resulting from reactions conducted at room temperature and the established optimal temperature of 37 °C. As shown in Figure 3e,f, a noticeable discrepancy in fluorescent intensity emerged within only 10 min. Following the 30 min reaction period, the average fluorescent value for the 37 °C reaction more than doubled the room temperature reaction. The visual contrast in the fluorescence was highly apparent after 30 min. Therefore, we confirmed that the optimum concentrations of the CRISPR components in our GO-CRISPR system were 120 nM for Cas12a and 140 nM for crRNA, with the *trans*-cleavage reaction executed at the established optimal temperature of 37 °C.

### Analytical Performance of *Salmonella* DNA Detection

**Figure 4:**
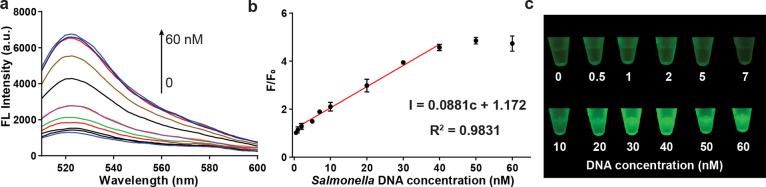
Detection of *Salmonella* DNA. (a) Fluorescence spectra of varying concentrations of DNA. (b) Ratio of fluorescent intensity values to the background at 520 nm for various concentrations of DNA, with linear regression for the dynamic range. (c). Fluorescent image of the GO-CRISPR system for the detection of *Salmonella* DNA at varying concentrations.

We subsequently aimed to evaluate the analytical performance of the GO-CRISPR system for the detection of *Salmonella* DNA. *Salmonella* DNA was first amplified by PCR and then diluted to various quantities. These DNA concentrations (0–60 nM) were then combined with optimized amounts of Cas12a, crRNA, and the ssDNA-FAM probe. Upon mixing, the enzyme, crRNA, and target formed activated Cas12a-crRNA complexes, exhibiting rapid *trans*-cleavage capabilities.^45^ A higher DNA concentration facilitated the rapid activation of a greater quantity of Cas12a-crRNA complexes, as illustrated in Figure 4a. The fluorescent signal prominently increases with an increase in DNA concentrations, becoming visually striking at or above 10 nM. As shown in Figure 4b, the fluorescent signal-to-background ratio increased linearly as the concentration increased to 40 nM, beyond which the increase plateaued. Therefore, it is likely that 40 nM DNA activated nearly all available Cas12a-crRNA complexes, reaching maximum probe degradation. The linear detection range (LDR) was determined to be between 2 and 40 nM, with a regression equation described as *I* = 0.0881*c* + 1.172 (*R*^2^ = 0.9831), where *I* represents the fluorescent intensity over the background and c indicates the concentration of *Salmonella* DNA. The limit of detection (LOD) was calculated to be 6.0 nM (LOD = 3.3*S*_*y*_/*S*, where *S*_*y*_ represents the standard deviation of the response from the negative control, and *S* represents the slope). Additionally, the fluorescent reaction was extremely apparent, beginning at 10 nM (Figure 4c). Considering the exponential amplification nature of RPA, these results indicate more than sufficient sensitivity when paired with upstream isothermal amplification.^47^

### Detection Sensitivity and Specificity of Bacteria

**Figure 5:**
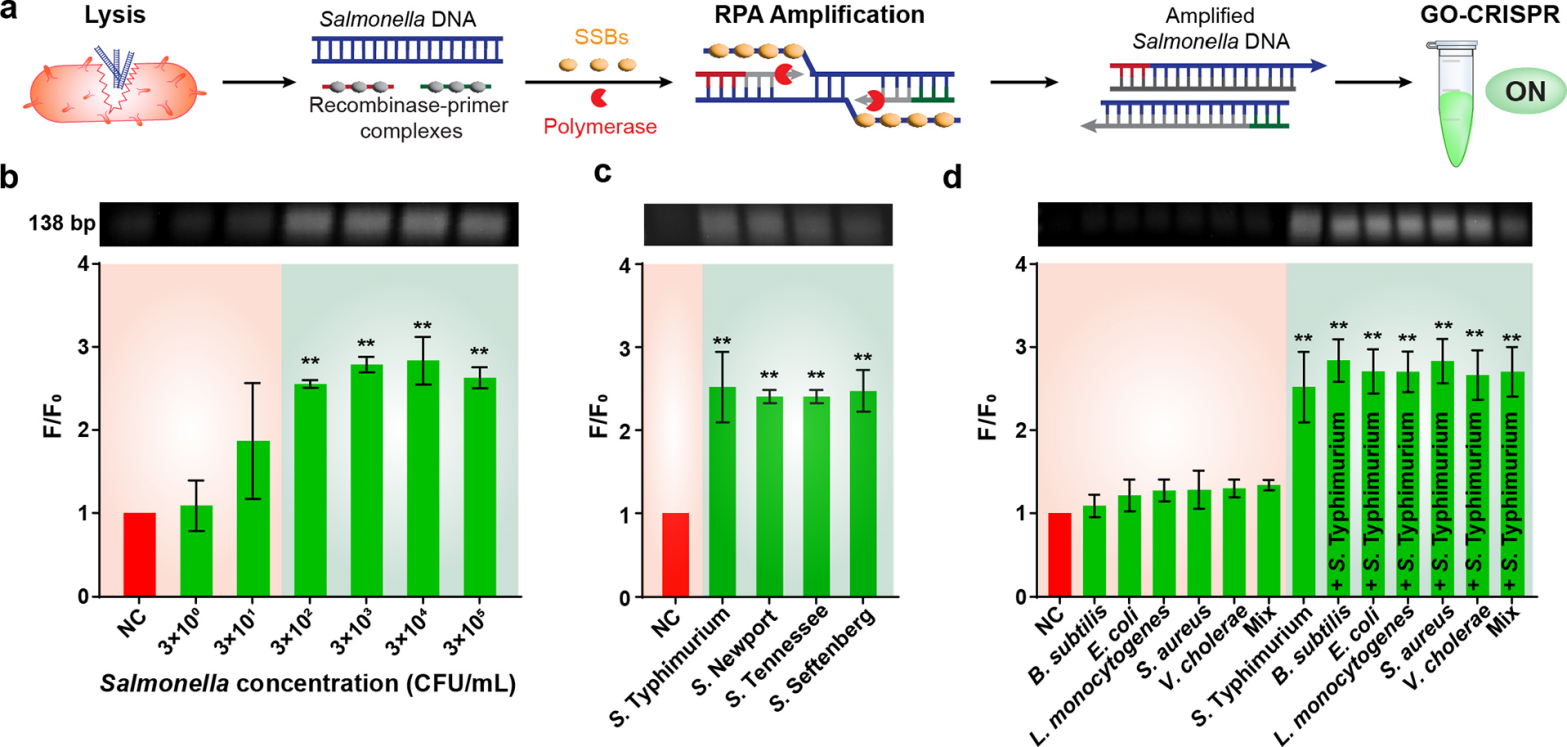
Detection sensitivity and specificity of the GO-CRISPR system for *Salmonella*. (a) Schematic illustration of RPA amplification and the GO-CRISPR system for *Salmonella*-specific detection. (b) Gel image of RPA products and fluorescent intensity of *S*. Typhimurium at various concentrations (NC = negative control). (c) Gel image of RPA products and fluorescent intensity of *Salmonella* serovars. (d) Gel image of RPA products and fluorescent intensity of competing bacteria strains (*B. subtilis*, *E. coli* O157:H7, *L. monocytogenes*, *S. aureus*, *V. cholerae*, and a “mix” of all five cultures) with and without *S*. Typhimurium. ^**^p, 0.05, Student’s unpaired *t* test.

We next explored the detection sensitivity and specificity of *S*. Typhimurium was detected using our GO-CRISPR assay. Following bacterial lysis at 95 °C for 15 min, genomic DNA was underwent RPA amplification. As illustrated in Figure 5a, the recombinase binds to primers, forming a complex capable of recognizing and displacing the template nucleic acids. Subsequently, an ssDNA binding protein (SSB) binds and stabilizes the displaced strand. This cyclic system facilitates exponential amplification of *Salmonella* DNA within 25 min, with gel electrophoresis results shown in Figure 5b–d.^48^*S*. Typhimurium (0–3 × 10^5^ CFU/mL) were lysed, amplified, and analyzed using our GO-CRISPR system (Figure 5b). Upon analysis of three repeated trials, concentrations containing 3 × 10^2^ or greater CFU/mL of *Salmonella* consistently yielded a fluorescent signal nearly three times greater than the background fluorescence. Notably, one of the trials containing 3 × 10^1^ CFU/mL also exhibited this desired fluorescent output, while the other two did not. This variability can be attributed to the chance-like nature of exponential amplification at lower DNA concentrations.^49^ Consequently, the LOD for *S*. Typhimurium was determined to be 3 × 10^2^ CFU/mL using Student’s unpaired *t*-test analysis. This level of sensitivity aligns with or surpasses that reported for similarly reported on-site biosensors for *Salmonella* detection.^50−52^

To evaluate the specificity of our assay, we conducted a comparative analysis with closely related bacterial strains. The evaluation encompassed four distinct serovars (*S*. Typhimurium, *S.* Newport, *S*. Tennessee, and *S.* Seftenberg). We leverage the highly conserved region of the *invA* gene target, which is anticipated to be present in as high as 99% of *Salmonella* strains.^53^ As illustrated in Figure 5c, the GO-CRISPR assay exhibited a remarkably high fluorescence signal-to-background ratio across all four analyzed serovars. Leveraging the highly conserved region of the *invA* gene target, our system demonstrates proficiency in detecting a substantial portion of *Salmonella* serovars, including the most prevalent *S*. Typhimurium. Therefore, our system demonstrated proficiency in detecting all four serovars tested, including the most prevalent *S*. Typhimurium.

We then evaluated our sensor for nonspecific pathogen detection. We analyzed *B. subtilis*, *E. coli* O157:H7, *L. monocytogenes*, *S. aureus*, and *V. cholerae* with our GO-CRISPR sensor. Additionally, a bacterial cocktail comprising all aforementioned nonspecific pathogens was examined. To investigate possible interference, we introduced *S*. Typhimurium into each mix of competing bacterial strains as well. Furthermore, no instances of nonspecific DNA amplification or CRISPR activity were observed, as all nonspecific bacteria yielded a fluorescent signal similar to the background (Figure 5d). The introduction of competing bacteria to *S*. Typhimurium did not hinder the amplification or the fluorescent signal. Notably, the mix of all nonspecific pathogens produced a visually dimmer amplification band. However, the fluorescent signal remained uncompromised in the presence of several competing bacteria. Moreover, the interference from nonspecific bacteria did not diminish the fluorescent output of our GO-CRISPR system. These results affirm that our RPA primers were designed with excellent specificity to *Salmonella*, and our CRISPR system exhibited exemplary specificity for the targeted *Salmonella* detection.

### Detection of *Salmonella* in Human Serum

**Figure 6:**
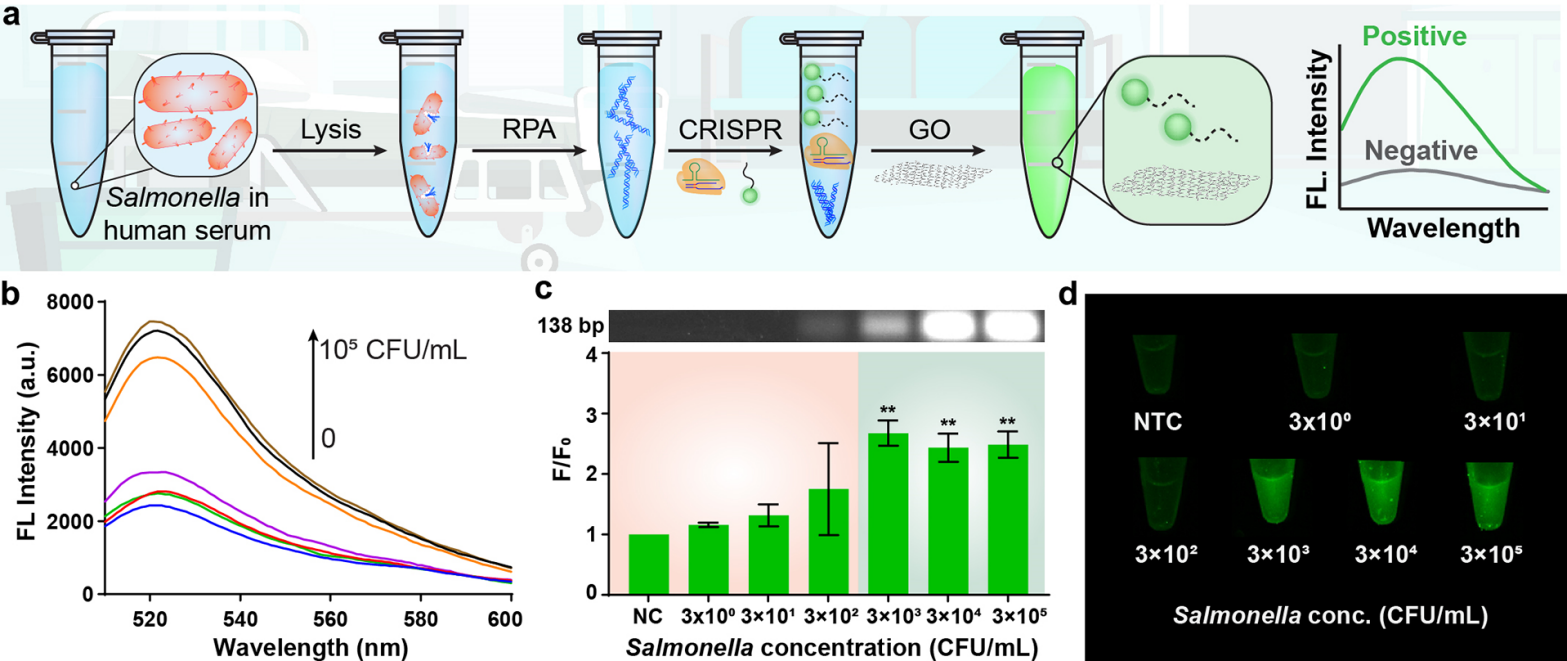
*Salmonella* detection in human serum. (a) Schematic illustration of the entire GO-CRISPR process for the detection of *Salmonella* in human serum. (b) Fluorescent spectra of varying concentrations of *S*. Typhimurium spiked in human serum. (c) Gel image of RPA products and the fluorescence intensity of varying concentrations of *S*. Typhimurium spiked in human serum. (d) Fluorescent image of varying concentrations of *S*. Typhimurium spiked in human serum ^**^p, 0.05, Student’s unpaired *t* test.

To ascertain the efficacy of the GO-CRISPR sensor in early sepsis detection, human serum was deliberately spiked with varying concentrations of *S*. Typhimurium (0–3 × 10^5^ CFU/mL), and subsequent analyses were conducted using our GO-CRISPR system (Figure 6a). Human serum utilizes the fluid component of blood, allowing us to analyze the inhibition of DNA amplification, CRISPR performance, and fluorescent readout from the complicated sample matrix. This enables us to assess the biosensor’s applicability for detecting bacterial infections in hospital settings. As shown in Figure 6b,c, the GO-CRISPR system reliably detected concentrations as low as 3 × 10^3^ CFU/mL in three repeated trials. This outcome was anticipated, given that the human serum underwent a 10-fold dilution in PBS after spiking with *Salmonella*. This was necessary to enhance DNA amplification while minimizing downstream interference in the fluorescent assay. A marginal increase in all fluorescence signals was observed, likely attributed to the presence of hormones, antibodies, and other proteins interfering with the noncovalent binding reaction between the GO and the ssDNA probe. However, the positive fluorescent signals were also enhanced, resulting in a fluorescent signal approximately 2.5 times higher than the background for *Salmonella* concentrations of 3 × 10^3^ CFU/mL or greater. The visual fluorescence readout was noticeably apparent for these samples in comparison to the negative control (Figure 6d). Therefore, we assert that our GO-CRISPR biosensor demonstrates satisfactory sensitivity for bacterial detection in blood samples for early sepsis detection.

## Conclusions

In conclusion, we developed a rapid and reliable sensing method for the on-site detection of sepsis-inducing bacteria. When combined with RPA amplification, we demonstrated that our GO-CRISPR biosensor can detect as little as 3 × 10^3^ CFU/mL of *Salmonella* in human serum. Furthermore, our system is highly specific for the target bacteria. The reported level of sensitivity and specificity is sufficient for bacterial detection within hospitals, which is crucial for sepsis prevention. Additionally, our sensor is truly rapid and point-of-care, providing a fluorescent readout without expensive or elusive technology in under an hour. The total time from sample collection to signal readout is under 1.5 h. Considering the dangers that quickly materialize from bacterial infections leading to sepsis, our GO-CRISPR system offers a vastly improved ability for early bacteria detection compared to traditional culture-based plate counting methods. The affordability and timeliness are especially attractive for resource-poor areas, where sepsis is the most prevalent. While our system was designed specifically for *Salmonella*, we envision that this system can be extended with multiple crRNAs in the same system to incorporate all of the most common sepsis-inducing bacteria including *E. coli* or *S. aureus*.^54^ This can create a personalized detection system owing to the supremely specific and tunable nature of CRISPR-based detection systems. Therefore, we believe that our sensor offers superior rapidity and simplicity for combating bacterial-induced sepsis in hospitals.

## Supporting Information Available

Oligonucleotide sequences, summary of CRISPR-based biosensors for bacteria detection, and the cost estimation of GO-CRISPR detection system (PDF)
