# Supplementary material for: On-Site Fluorescent Detection of Sepsis-Inducing Bacteria using a Graphene-Oxide CRISPR-Cas12a (GO-CRISPR) System
Source: Anal Chem. 2024 Jan 30;96(6):2676–83. doi: 10.1021/acs.analchem.3c05459 (PMC10867801; doi:10.1021/acs.analchem.3c05459)
Supplement: Supplementary file 1 — ac3c05459_si_001.pdf [file ac3c05459_si_001.pdf]

Supporting Information

On-site Fluorescent Detection of Sepsis-Inducing Bacteria using a Graphene-Oxide CRISPR-Cas12a (GO-CRISPR) System

Tom Kasputis[1], Yawen He[1], Qiaoqiao Ci[1], and Juhong Chen[1,2]*

[1]Department of Biological Systems Engineering, Virginia Tech, Blacksburg, VA, 24061, United States

[2]Department of Bioengineering, University of California, Riverside, CA, 92521, United States

*Corresponding author. jchen@ucr.edu

Table of Contents

1. Oligonucleotide sequences
2. Summary of the performance of CRISPR-based detection of bacterial pathogens
3. Cost analysis of the fluorescent GO-CRISPR detection system
4. References

Table S1. Oligonucleotide sequences

DNA Name	Sequence (5'-to-3')
ssDNA-FAM-Probe	**56-FAM-**TATGCTCTTTAAAAAAAAAAAAAAAAAAAA
Salmonella-RPA-F	TGTTGTCTTCTCTATTGTCACCGTGGTCCAG
Salmonella-RPA-R	CATCTGTTTACCGGGCATACCATCCAGAGAAAA
Salmonella-PCR-F	CTATTGTCACCGTGGTCCAGTTTATCGTTATTA
Salmonella-PCR-R	CCAATAAAGTTCACAAAGATAATAATGATGCC
Salmonella-crRNA	UAAUUUCUACUCUUGUAGAUCCGGGCAUACCAUCCAGAGAAAA

Table S2. Summary of the performance of CRISPR-based detection of bacterial pathogens

Method	Amplification method	Bacterial Target	Limit of Detection	Application
Cas12a logic gates[1]	PCR	*S. aureus*	10^3 CFU/mL	Milk samples
Cas12a-CPA[2]	PCR	*N. farcinica*	10^5 CFU/mL	Sputum specimens
Cas12a electrochemical[3]	RAA	*L. monocytogenes*	2.6×10^2 CFU/mL	Spiked *F. velutipes*
Cas12a FQ probe[4]	RPA	*M. tuberculosis*	50 CFU/mL	Clinical samples
Digital droplet CRISPR-Cas12[5]	LAMP	*Salmonella*	10^2 CFU/mL	Milk samples
GO-CRISPR Cas12a	RPA	*Salmonella*	3×10^2 CFU/mL	Human serum

Table S3. Cost analysis of the fluorescent GO-CRISPR detection system

Material	Cost per Reaction
Cas12a enzyme	$1.90
crRNA	$0.20
FAM probe	$0.01
Single-layer graphene oxide	< $ 0.01
**Total**	**$2.11**

References

(1) Peng, L.; Zhou, J.; Yin, L.; Man, S.; Ma, L. Integration of logic gates to CRISPR/Cas12a system for rapid and sensitive detection of pathogenic bacterial genes. *Analytica Chimica Acta* **2020**, *1125*, 162-168. DOI: https://doi.org/10.1016/j.aca.2020.05.017.

(2) Qiu, X.; Xu, S.; Liu, X.; Han, L.; Zhao, B.; Che, Y.; Han, L.; Hou, X.; Li, D.; Yue, Y.; et al. A CRISPR-based nucleic acid detection platform (CRISPR-CPA): Application for detection of Nocardia farcinica. *Journal of Applied Microbiology* **2022**, *132* (5), 3685-3693. DOI: 10.1111/jam.15424.

(3) Li, S.-Y.; Cheng, Q.-X.; Wang, J.-M.; Li, X.-Y.; Zhang, Z.-L.; Gao, S.; Cao, R.-B.; Zhao, G.-P.; Wang, J. CRISPR-Cas12a-assisted nucleic acid detection. *Cell Discovery* **2018**, *4* (1). DOI: 10.1038/s41421-018-0028-z.

(4) Ai, J.-W.; Zhou, X.; Xu, T.; Yang, M.; Chen, Y.; He, G.-Q.; Pan, N.; Cai, Y.; Li, Y.; Wang, X.; et al. CRISPR-based rapid and ultra-sensitive diagnostic test for *Mycobacterium tuberculosis*. *Emerging Microbes & Infections* **2019**, *8* (1), 1361-1369. DOI: 10.1080/22221751.2019.1664939.

(5) Wu, H.; Cao, X.; Meng, Y.; Richards, D.; Wu, J.; Ye, Z.; deMello, A. J. DropCRISPR: A LAMP-Cas12a based digital method for ultrasensitive detection of nucleic acid. *Biosensors and Bioelectronics* **2022**, *211*, 114377. DOI: https://doi.org/10.1016/j.bios.2022.114377.
